# Draft Genome Sequences of *Acinetobacter parvus* CM11, *Acinetobacter radioresistens* CM38, and *Stenotrophomonas maltophilia* BR12, Isolated from Murine Proximal Colonic Tissue

**DOI:** 10.1128/genomeA.01089-15

**Published:** 2015-10-15

**Authors:** Azadeh Saffarian, Céline Mulet, Tomoaki Naito, Christiane Bouchier, Magali Tichit, Laurence Ma, Gianfranco Grompone, Philippe J. Sansonetti, Thierry Pédron

**Affiliations:** a Unité de Pathogénie Microbienne Moléculaire, INSERM U1202, Institut Pasteur, Paris, France; b Chaire de Microbiologie et Maladies Infectieuses, Collège de France, Paris, France; c Yakult Central Institute, Yakult Honsha Co., Ltd, Izumi, Kunitachi-shi, Tokyo, Japan; d Plate-forme Génomique, Département Génomes et Génétique, Institut Pasteur, Paris, France; e Microbiota Unit, Bioaster, Paris, France

## Abstract

Here, we report three genome sequences of bacteria isolated from murine proximal colonic tissue and identified as *Acinetobacter parvus* CM11, *Acinetobacter radioresistens* CM38, and *Stenotrophomonas maltophilia* BR12.

## GENOME ANNOUNCEMENT

Recently, by laser capture microdissection and pyrosequencing, we identified bacteria located inside murine proximal colonic crypts (1). These bacteria were aerobic, nonfermentative, and mainly belong to the genus *Acinetobacter*. *Delftia* and *Stenotrophomonas* spp. were also identified. In order to study the impact of these bacteria on the intestinal epithelium, the first step was to cultivate them. Proximal colonic tissues from C57BL/6 mice (Elevage Janvier) were washed with bleach and homogenized in 2 ml of sterile phosphate-buffered saline (PBS) using the Precellys system with 2.8-mm ceramic beads. This mixture was then added to 30 ml of a minimum medium (2). The culture were incubated at 30°C for 48 h, with shaking at 300 rpm/min in a Multitron incubation shaker (Infors) and isolated on agar plates (GTCS, MacConkey, Herellea, and CHROMagar). Selected colonies were reisolated on CHROMagar to obtain a pure colony. The colonies were identified using the Biolog system with a GEN III MicroPlate. Strain identification was confirmed by Sanger sequencing of 16S, *rpoB*, and *gyrB* genes after genomic DNA extraction by the Wizard genomic DNA purification kit, according to the manufacturer’s instructions (Promega). The genomes were sequenced with the Illumina HiSeq 2000 technology (paired-end libraries) at the Institut Pasteur of Paris, France.

The numbers of paired-end reads for all samples are indicated in Table 1. For *Acinetobacter parvus* CM11 and *Acinetobacter radioresistens* CM38, the average base quality for Read 1 and Read 2 is >30, and for *Stenotrophomonas maltophilia* BR12, it is >32 and 20, respectively. The 100-base reads were filtered out using Prinseq-lite 0.20.3 (3).

**TABLE 1:** Characteristics of the three genomes

Species	Length of genome (Mb)	G+C content (%)	No. of paired-end reads		No. of contigs obtained from assembly *de novo* with CLC Assembler	Avg length of contigs (bp)	Total of assembled contigs (Mb)	*N*_50_ (bp)	No. of contigs after cleaning	Total of bases after cleaning (Mb)	RAST annotation results		Accession no.^a^
			Raw data	After filtering using Prinseq-lite							No. of coding sequences	No. of genes involved in resistance to antibiotics and toxic compounds	
*A. parvus* CM11	3.34	38.70	37,132,842	9,039,763	972	4,511.26	4.38	213,844	118	3.45	3,259	45	LACJ00000000
*A. radioresistens* CM38	3.14	41.50	29,971,045	7,018,315	51	62,364.14	3.18	165,532	41	3.17	2,974	39	LATS00000000
*S. maltophilia* BR12	4.85	66.30	57,576,810	2,864,668	94	46,920.68	4.43	92,072	79	4.4	3,905	49	LATR00000000

a The contigs are merged into a single accession number.

The parameters of filtering were fixed to remove all reads with a quality score of <30 for bases and/or an average quality score of <35 for read sequences (for *A. parvus* CM11 and *A. radioresistens* CM38) and <33 for *S. maltophilia* BR12. Exact or a reverse complementary duplicate (for both R1 and R2), reads containing ambiguous base N for >1% of their length, and reads of low complexity (entropy value, <70) were removed. The numbers of paired-end reads remaining after filtering are given in Table 1. Those reads have been used for producing the contigs using the assembly *de novo* option of CLC-Assembler 4.2.0 and CLC Genomics Workbench (CLC bio).

Moreover, the contigs were cleaned: all contigs <1,000 bases and/or containing the ambiguous base N for >15% of their length were removed. The numbers of contigs before and after cleaning are shown in Table 1.

Functional annotation was carried out on cleaned contigs using tools from RAST (4, 5) (http://rast.nmpdr.org/) and from the MicroScope platform (6) (http://www.genoscope.cns.fr/agc/microscope/mage/). RAST annotation (Table 1) showed the genes involved in antibiotic resistance, and interestingly, the gene encoding the β-lactamase was present in the three strains. The knowledge of the complete sequence of these strains will allow us to better understand the impact of these bacteria on the intestinal epithelium.

### Nucleotide sequence accession numbers.

The draft genome sequences have been deposited at DDBJ/EMBL/GenBank under the accession numbers listed in Table 1.
